# Systematic review and meta-analysis of hepatitis C virus infection and HIV viral load: new insights into epidemiologic synergy

**DOI:** 10.7448/IAS.19.1.20944

**Published:** 2016-09-19

**Authors:** Nicholas Petersdorf, Jennifer M Ross, Helen A Weiss, Ruanne V Barnabas, Judith N Wasserheit, for the HCV and HIV Transmission Working Group

**Affiliations:** 1 London School of Economics, University of London, London, UK; 2 Division of Infectious Diseases, University of Washington, Seattle, WA, USA; 3 MRC Tropical Epidemiology Group, Department of Infectious Disease Epidemiology, London School of Hygiene and Tropical Medicine, University of London, London, UK; 4 Department of Epidemiology, University of Washington, Seattle, WA, USA; 5 Department of Global Health, University of Washington, Seattle, WA, USA

**Keywords:** hepatitis C virus, HCV, HIV, co-infection, systematic review, viral load

## Abstract

**Introduction:**

Hepatitis C virus (HCV) and HIV infection frequently co-occur due to shared transmission routes. Co-infection is associated with higher HCV viral load (VL), but less is known about the effect of HCV infection on HIV VL and risk of onward transmission.

**Methods:**

We undertook a systematic review comparing 1) HIV VL among ART-naïve, HCV co-infected individuals versus HIV mono-infected individuals and 2) HIV VL among treated versus untreated HCV co-infected individuals. We performed a random-effects meta-analysis and quantified heterogeneity using the *I*^2^ statistic. We followed Cochrane Collaboration guidelines in conducting our review and PRISMA guidelines in reporting results.

**Results and discussion:**

We screened 3925 articles and identified 17 relevant publications. A meta-analysis found no evidence of increased HIV VL associated with HCV co-infection or between HIV VL and HCV treatment with pegylated interferon-alpha-2a/b and ribavirin.

**Conclusions:**

This finding is in contrast to the substantial increases in HIV VL observed with several other systemic infections. It presents opportunities to elucidate the biological pathways that underpin epidemiological synergy in HIV co-infections and may enable prediction of which co-infections are most important to epidemic control.

## Introduction

Approximately 2.3 million people worldwide are infected with both HIV and hepatitis C virus (HCV) [1]. HIV infection is associated with increased HCV replication, and co-infected patients have higher HCV viral load (VL) compared to their mono-infected counterparts [2]. Additionally, the risk of mother-to-child transmission of HCV infection is nearly twofold higher in HIV-HCV co-infected women than in HCV mono-infected women. Furthermore, the risk of cirrhosis or liver failure is higher in co-infected individuals as compared to those with either HCV or HIV mono-infection [3,4]. However, less is known about the effect of HCV on HIV VL and onward transmission. Other HIV co-infections such as acute malaria, herpes simplex 2, and tuberculosis increase HIV VL [5], likely through inflammatory-mediated mechanisms. HIV VL is the key determinant of HIV transmission [6,7] and, consequently, these HIV co-infections have the potential to increase the probability of HIV transmission through significant increases in VL.

New HCV treatment options provide opportunities for sustained virological response over a short duration of therapy (8 to 12 weeks) with a high success rate (>90%), including among HIV co-infected persons [8–11]. However, the majority of the 130 to 150 million people living with HCV worldwide are unaware of their infection and do not have access to treatment [12–14]. If HCV co-infection increases HIV VL and consequently HIV transmission and progression, HCV diagnosis and treatment programmes could potentially decrease HIV incidence at the population level, in addition to decreasing HCV incidence and prevalence. HCV treatment programmes that do not account for this additional health benefit would underestimate the cost-effectiveness of HCV treatment.

Results of studies looking at the effect of HCV co-infection on HIV VL are mixed. Some observational studies reported higher HIV VL in co-infected persons [15], whereas others found no effect on HIV VL [16], and still others reported lower HIV VL in co-infected persons [17]. Notably, the majority of studies have been conducted in populations being treated with antiretroviral therapy (ART). A recent systematic review and meta-analysis of the impact of HCV on immunological and virological responses after ART initiation suggested an adverse effect of HCV on immune recovery of HIV-infected individuals initiating ART, especially among those with initially impaired immunological status (CD4 count below 350 cells/mm^3^) [18]. To our knowledge, the effect of HCV co-infection on HIV VL among ART-naïve individuals has not been systematically reviewed. In low- and middle-income countries, nearly 60% of the 34.4 million people living with HIV lack access to ART [19]. Therefore, understanding the impact of HCV and its treatment on HIV infection is particularly relevant in this underserved population.

We hypothesize that HCV co-infection increases HIV VL through inflammatory-mediated mechanisms. To address this question, we reviewed the evidence and conducted a meta-analysis for an association of HCV co-infection and treatment with HIV-related outcomes among ART-naïve patients.

## Methods

We followed Cochrane Collaboration guidelines in conducting our review [20] and PRISMA guidelines in reporting results [21].

### Study objectives and criteria for considering studies for this review

The six study objectives were to assess the impact of 1) untreated HCV infection on HIV-1 VL; 2) HCV treatment on HIV-1 VL; 3) untreated HCV infection on HIV-1 transmission in HIV-1 serodiscordant couples; 4) HCV treatment on HIV-1 transmission in HIV-1 serodiscordant couples; 5) untreated HCV infection on HIV-1 acquisition; and 6) HCV treatment on HIV-1 acquisition.

Observational epidemiological studies and randomized controlled trials were eligible. Studies that included the following participants were excluded from the review: participants aged 12 years or younger, patients on HIV treatment, pregnant women and HIV-2 infected patients. In addition, patients on HCV treatment were excluded from Objectives 1, 3 and 5. For studies in which a subgroup of participants did not meet the inclusion criteria, results were extracted for eligible participants if studies allowed for this (e.g. if results were stratified by ART status).

### Search strategy for identification of studies

Electronic searches of the PubMed and Embase databases were conducted on 30 July 2013 and updated on 11 February 2015. The following search was conducted in PubMed: ((HCV [MeSH Terms]) OR HCV [Text Word] OR HCV [Text Word]) AND (human immunodeficiency virus [MeSH Terms] OR human immunodeficiency virus [Text Word] OR HIV [Text Word] OR acquired immune deficiency syndrome [MeSH Terms]) AND (“Epidemiologic Studies” [MeSH] OR “clinical trials as topic” [MeSH]).

A similar search that excluded articles indexed in PubMed was conducted in Embase using the following search terms: ((“hepatitis”/exp OR “hepatitis”/de AND c AND (“virus”/exp OR “virus”/de)) OR hcv) AND ((“human”/exp OR “human”/de) AND (“immunodeficiency”/exp OR “immunodeficiency”/de) AND (“virus”/exp OR “virus”/de)) OR “HIV”/exp OR “HIV”/de OR (acquired AND immune AND deficiency AND (“syndrome”/exp OR “syndrome”/de)) NOT [medline]/lim AND ([adolescent]/lim OR [adult]/lim OR [aged]/lim) AND [humans]/lim AND [embase]/lim. Search results were collated using the reference manager EndNote (Thomson Reuters, New York, NY, USA) to identify any remaining duplicate citations.

The searches included all languages and were limited to studies in humans. The search was not restricted by specified start or end dates and ran through the date on which the updated search was conducted on 11 February 2015. Reference lists of papers meeting criteria were hand searched for additional articles. Abstracts were reviewed from the past three meetings of the Conference on Retroviruses and Opportunistic Infections, the International Symposium on HCV and Related Viruses, ID Week/Infectious Disease Society of America, the International Liver Congress/EASL and the Liver Meeting/AASLD.

### Selection of studies, data extraction and synthesis

Abstracts were reviewed and full-text articles of potentially relevant studies were reviewed independently by two authors (NEP and RVB, HAW or JNW) against predefined criteria. Data were extracted by NEP and JMR, using a standardized data extraction form. Discrepancies were discussed and consensus reached. Authors were contacted for papers that reported that they had gathered data of interest but did not present these data in the text or did not completely report all results and statistics.

When data from the same individuals were reported in multiple publications, we used the publication that incorporated the most relevant data or most comprehensive analysis. Publications with overlapping cohorts were considered on a case-by-case basis. We estimated CIs based on standard deviations reported or displayed in the articles.

The methodological quality of included studies was reviewed by NEP and JMR using a tool to record the confounding variables assessed in the study. Discrepancies in quality rating were discussed and consensus reached. Studies were rated as low, moderate or high risk of bias. A key factor was how studies accounted for time since HIV seroconversion, because VL is dynamic and changes over time. Adjustment for CD4 and sociodemographic variables was also considered. If these factors were handled appropriately in the design and analysis, studies that reported HIV VL stratified by CD4 count were generally rated as low risk of bias. In contrast, studies that reported and compared the mean CD4 counts of each group were rated as medium risk. Studies that did not adjust for CD4 count were rated as high risk.

### Statistical methods

The primary outcome of interest was the mean log_10_ HIV VL difference between HIV-HCV co-infected and HIV mono-infected individuals for Objective 1 and by HCV treatment status for Objective 2. Meta-analysis was performed using the DerSimonian and Laird method for random effects and implemented with the “metan” command in Stata. Heterogeneity was quantified using the *I*^2^ statistic. Funnel plots were visually examined and Egger tests were conducted to assess the possibility of publication bias. All statistical analyses were conducted in STATA version 13 (StataCorp, College Station, TX, USA).

## Results and discussion

**Figure 1:**
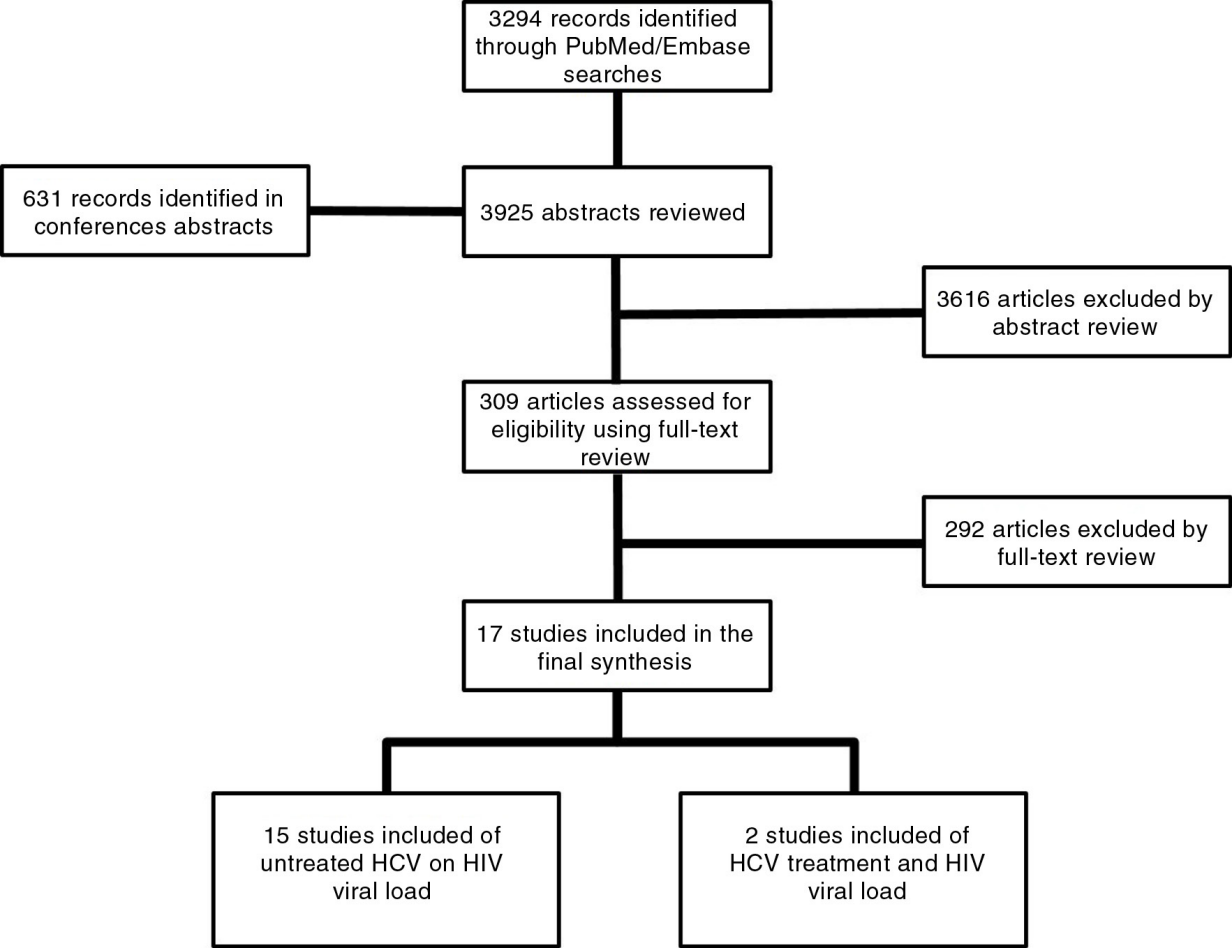
PRISMA flow diagram of studies included in systematic review.

A total of 17 studies were included out of 3925 articles and abstracts that were screened (Figure 1). Overall, the three most common reasons for exclusion were the patients having received ART, inappropriate comparison group and primary outcomes that were not relevant for this analysis. Of the 17 articles identified, 15 addressed the impact of HCV infection on HIV VL (Objective 1) [4,15–17,22–32], whereas two studies [33,34] examined the impact of HCV treatment on HIV VL (Objective 2). Because no studies were identified that addressed the other four objectives, this article describes results of the review and meta-analysis of Objectives 1 and 2.

### Objective 1: Impact of HCV infection on HIV viral load

Details of the 15 studies eligible for Objective 1 are shown in Table 1. The studies were conducted in China, Europe, Africa and North America. Two studies were cross-sectional [25,32], whereas the remainder were longitudinal in design (nine prospective [4,15,17,22,23,26,27,30,31], three retrospective [16,28,29] and one case control [24]). The quality of the studies varied, and adjustment for confounders was conducted in only five studies. Three of the studies were assessed as low risk of bias [22,23,28], three with a medium risk [4,16,26] and the remainder with a high risk of bias [15,17,24,25,27,29–32]. Data included in the meta-analysis evaluated HIV VL at a single time point. The studies ranged in size from 16 [24] to 1510 [28] participants.

**Table 1:** Studies included in the systematic review for Objective 1

First author	Data collection	Location	Study design	Population	HIV/HCV co-infected^a^		HIV mono-infected	
	
					*N*	Mean log_10_ HIV viral load (95% CI)^b^	*N*	Mean log_10_ HIV viral load (95% CI)^b^
Zhang	1992 to 1995	China	Retrospective cohort^c^	Former blood donors	135	4.15 (4.05 to 4.25)	20	4.43 (4.18 to 4.68)
Li	NA	China	Prospective cohort^c^	Research centres	58	4.43 (3.71 to 5.15)	117	4.47 (3.83 to 5.11)
Liang	1994 to 2006	Taiwan	Retrospective cohort^d^	IDUs at referral hospital	79	4.55 (4.40 to 4.70)	9	5.04 (4.61 to 5.47)
Filippini	1996 to 2001	Italy	Prospective cohort^d^	HIV-infected persons	24	4.84 (2.76 to 6.92)	36	4.45 (2.78 to 6.11)
Filippini	NA	Italy	Case control		8	4.50 (4.01 to 4.99)	8	4.26 (4.07 to 4.45)
Antonucci	1997 to 2004	Italy	Prospective cohort^d^	Italian Cohort Naïve for Antiretrovirals	156^e^	4.77 (2.60 to 6.53)^f^	1219	4.91 (1.30 to 6.88)^f^
Braitstein	1996 to 2003	Canada	Retrospective cohort^c^	HIV/AIDS Drug Treatment Programme	606	4.74 (4.70 to 4.77)	580	4.72 (4.68 to 4.76)
Rockstroh	1994 to 2003	Argentina, Europe, Israel	Prospective cohort^d^	EuroSIDA cohort	202	4.26 (4.13 to 4.38)	428	4.23 (4.14 to 4.32)
Kovacs	1994 to 1995	USA	Prospective cohort^c^	Women's Interagency HIV Study	215^g^	3.92 (3.80 to 4.04)	331	4.05 (3.95 to 4.15)
Cheng	NA	USA	Prospective cohort^c^	People with alcohol problems living with HIV	100	2.59 (2.28 to 2.90)	110	2.82 (2.31 to 3.11)
Körner	NA	Germany	Cross sectional		14	4.66 (2.65 to 5.74)	25	4.60 (1.70 to 5.58)
Sullivan	1998 to 2004	USA	Prospective cohort^c^	Adult and Adolescent Spectrum of HIV Disease	269	4.93 (4.86 to 5.00)	1241	4.92 (4.88 to 4.95)
Lodwick	1990 to 2004	Europe	Prospective cohort^c^	Swiss HIV Cohort Study with CD4 > 350^h^	569	3.97 (3.87 to 4.07)	605	4.23 (4.15 to 4.31)
Greub	1996 to 2000	Europe	Prospective cohort^c^	Swiss HIV Cohort Study	476	4.64 (4.55 to 4.73)	932	4.62 (4.56 to 4.68)
Salpini	2010	Cameroon	Cross sectional	HIV-infected persons	18	4.63 (3.99 to 5.27)	52	4.60 (4.24 to 4.96)

a HCV test by antibody only for Zhang, Li, Filippini 2003, Rockstroh, Lodwick, Braitstein, Greub and Salpini; by HCV RNA for Liang, Filippini 2000, Antonucci, Kovacs, Cheng and Korner

b HIV RNA test by polymerase chain reaction (PCR) for Filippini 2000, Filippini 2003, Liang, Zhang, Greub and Salpini; by branched DNA (bDNA) for Li; by either PCR or bDNA for Cheng and Korner; by nucleic acid sequence based amplification for Kovacs; or method not specified in publication for Antonucci, Braitstein, Lodwick, Rockstroh and Sullivan

c data from follow-up

d data from baseline

e patients with an HCV RNA load above 1×106 IU/mL

f median and range

g includes patients with HCV viremia and aviremic patients

h cohort used in this analysis excludes patients who were included in the Greub study. HCV, hepatitis C virus; NA, not applicable.

The studies varied in whether they used the presence of HCV RNA or HCV antibody to classify participants’ HCV infection status. Three studies considered separately those who had a positive HCV antibody without detectable RNA versus those who were also viremic, finding that 12 to 18% of antibody-positive patients had undetectable HCV RNA [22,23,26]. Two studies only included co-infected participants with detectable HCV RNA [24,25]. One study tested for HCV RNA in subjects who were HCV antibody positive but did not clearly restrict its analysis to participants with HCV viremia [29]. Eight studies classified participants based on HCV antibody testing alone without RNA confirmation of active viremia [4,15–17,27,30–32]. The final study classified HCV infection based on diagnosis codes from the medical record [28]. None of the studies reported testing for HCV RNA in those participants with a negative HCV antibody.

Only four studies found strong evidence of association (*p<*0.05) between HIV-HCV co-infection and HIV VL, with all four pointing towards higher HIV VL among mono-infected patients than among co-infected patients [17,22,29,30]. The majority of the studies revealed no significant association.

**Figure 2:**
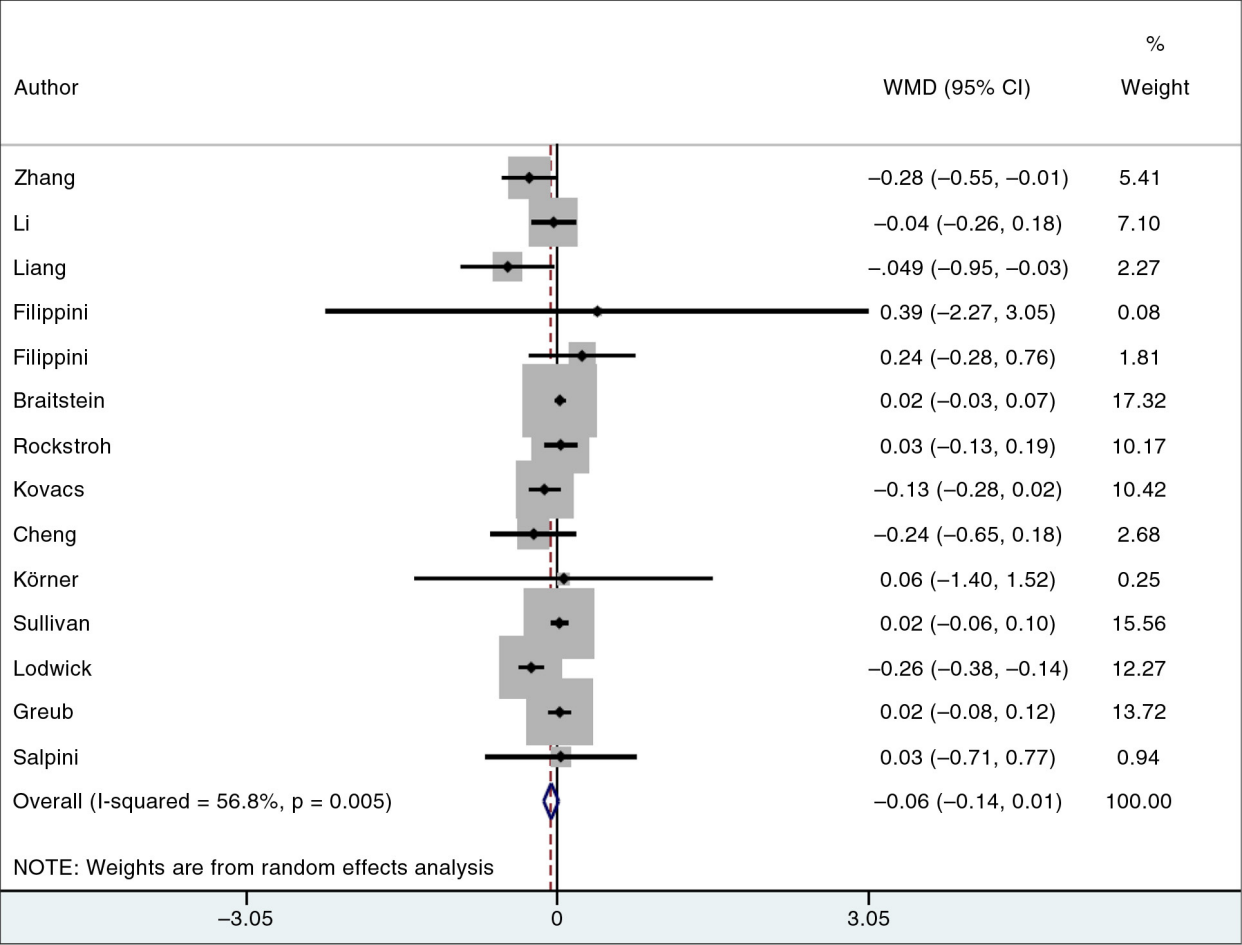
Random-effects meta-analysis of the mean difference in HIV viral load (log_10_ copies/mL) between HIV/HCV co-infected and HIV mono-infected individuals.

Fourteen of the fifteen studies were included in the meta-analysis (Figure 2) [4,15–17,23–32]. There was evidence of moderate heterogeneity in effect size between studies (*I*^2^=56.8%; *p=*0.005). The summary estimate of the mean difference between VL in the co-infected vs mono-infected was −0.06 log_10_ copies/mL (95% CI: −0.14, 0.01; *p=*0.09). The study not included in the meta-analysis was one in which only the median HIV VLs were given (4.77 log_10_ copies/mL in co-infected individuals with an HCV VL above 1×10^6^ IU per mL, 4.70 log_10_ copies/mL in co-infected individuals with an HCV VL 5.1×10^6^ IU per mL and 4.91 log_10_ copies/mL in mono-infected; *p=*0.01) [22].

A *post hoc* analysis of studies that only included patients with HCV viremia in the HIV-HCV co-infection arm [23–26,29] also found no difference in HIV VL between co-infected and HIV mono-infected patients (Figure 1; Supplementary file).

### Objective 2: Impact of HCV treatment on HIV viral load

Only two studies reported outcomes of interest for Objective 2 [33,34]. The studies found different effects of HCV therapy with pegylated interferon-alpha-2a/b and ribavirin on HIV VL. In the Obermeier *et al*. study, treatment was associated with an increased mean HIV VL of 4.52 log_10_ copies vs 4.14 log_10_ copies in untreated individuals (no confidence intervals provided). In the COHERE study, HIV VL decreased from 4.20 (95% CI (3.31, 5.09)) at enrolment in the study to 4.00 (95% CI (3.09, 4.91)) 12 weeks after the start of HCV treatment. However, both studies found that treatment with pegylated interferon-alpha-2a/b and ribavirin had no statistically significant impact on HIV VL (Obermeier *et al*.: *p*=0.61; COHERE: *p*=0.17).

## Discussion

The principal finding from this study is that patients co-infected with HIV and HCV do not have significantly higher HIV VL than patients with HIV who are HCV-uninfected. The subset of studies that restricted their analysis to patients with documented HCV viremia found either no significant (*p<*0.05) difference in HIV VL between HIV mono-infected and HIV/HCV co-infected participants [23–26] or significantly lower HIV VL in the co-infected population [22,29].

The finding is interesting for several reasons. First, patients co-infected with HIV and other pathogens, including herpes simplex virus, malaria and tuberculosis, exhibit significantly higher levels of HIV RNA in the blood [5]. Second, HCV infection does affect other markers of HIV disease progression such as inhibiting CD4 count recovery [18,35,36]. Third, the relationship between HIV and HCV VL is non-reciprocal: other studies have shown that HIV co-infection increases HCV VL in subjects by increasing the rate of viral replication [37] and immune suppression, as well as accelerating liver disease progression [3]. The surprising lack of association between HCV infection and HIV VL offers an opportunity to further explore the different mechanisms underpinning interactions between HIV and HCV compared with those underpinning interactions between HIV and other pathogens that result in persistent, systemic infections.

Co-infection with HIV and tuberculosis, malaria and herpes simplex virus results in higher HIV VL through multiple pathways that affect the equilibrium between HIV viral replication and control of the virus by the host immune system. Many efforts to understand HIV replication focus on the transcriptional promoter for the HIV-1 genome, which lies within the long terminal repeat that encodes the transactivation response element [38]. This promoter interacts with the viral transcriptional activator protein tat, which is necessary for viral replication [39,40] and operates in conjunction with several host transcriptional co-factors. Cytokines such as tumour necrosis factor alpha (TNF-α), interleukin (IL)-1β and IL-6 also affect viral replication [41]. Co-infecting pathogens increase HIV viral replication through de-repression of the transcriptional promoter (active tuberculosis) [42,43], activation by antigen presenting cells and increase in TNF-α secretion (malaria) [44–46], and transcriptional activation and cytokine release (HSV-2) [47,48].

The relationship between hepatitis B virus (HBV) infection and HIV VL demonstrates similarities with the mechanisms activated by the co-infecting pathogens described above. HBV X protein induces transcription of the HIV genome by synergizing with tat protein [49]. To our knowledge, the effect of HBV status on HIV VL has not been systematically reviewed, but observational studies in several settings indicate mixed results. Ladep and colleagues and Idoko and colleagues found a significantly higher HIV VL among ART-naïve Nigerian patients with HIV-HBV co-infection than among HIV mono-infected patients [50,51]. In contrast, Hoffmann and colleagues found no difference in HIV VL by HBV infection status in a large study of South Africans initiating ART [52].

We may expect HCV infection to increase HIV VL because, *in vitro*, HCV has been shown to induce transcription of the HIV-1 genome [53]. However, as we did not find an increased HIV VL among those with HCV co-infection, we propose several alternate hypotheses for evaluation. One hypothesis is that since the burden of HCV infection is in the liver, HCV may not be available to interact with HIV at the major site of HIV replication in the lymphoid tissues. Despite this, HCV infection has been reported to occur in the human peripheral blood mononuclear cells that are the primary site of HIV infection and in these sites is associated with increases in TNF-α and IL-8 [54,55]. Additionally, *in vitro*, endocytosis of HCV and HIV virions by blood monocytes or plasmacytoid dendritic cells can induce production of interferon [56] or IL-8 as well as activation of the NLRP3 inflammasome to produce and release IL1-β without requiring a productive infection of the cell itself [57]. A second hypothesis is that inflammasome induction by HCV may limit HIV replication in co-infected cells, which may mask an inflammation-induced increase in HIV VL occurring in cells without HCV infection (M Gale, pers. commun., 9 October 2015). A third hypothesis is that co-infecting pathogens such as herpes simplex virus that result in episodes of symptomatic or asymptomatic reactivation episodes or pathogens causing repeated bouts of infection, as with malaria, may cause more of a systemic inflammatory response. Finally, there may be characteristics of the HIV-1 strains associated with particular modes of transmission, such as intravenous drug use, that confound the relationship between HCV infection status and HIV VL [58].

Notably, study limitations may have precluded detection of a difference between HIV VL in mono-infected patients and those with HIV-HCV co-infection. The quality of the studies, including the methods used to control for potential confounders, varied substantially between the studies, and this is likely to have contributed to the statistical heterogeneity between study results. Some did not control for any potential confounders, while others controlled extensively for patient characteristics that might influence VL levels, including CD4+ counts, age, ethnicity, HIV-1 clade and mode of HIV transmission. Additionally, this systematic review found a dearth of high quality studies on the impact of HCV infection on HIV acquisition and transmission, as well as on the impact of HCV treatment on HIV infection, acquisition and transmission (Objectives 3 to 6). This is due in part to the fact that the search criteria excluded studies and patients experienced with ART for HIV, which was done because of the effectiveness of ART in decreasing HIV VL. As expected, very few studies from the mid-1990s onwards could be included because very few patients were ART-naïve. Even in the included studies that enrolled ART-naïve participants, the majority reported a single baseline HIV VL prior to ART administration. Thus, an additional limitation in the existing literature is that it does not permit conclusions about the interaction between HIV and HCV over time.

Bidirectional misclassification of active HCV status due to HCV antibody testing may have limited the ability of these studies to detect an effect of HCV infection on HIV VL. Whereas nearly half of the studies used HCV RNA testing to confirm active infection in patients who were antibody positive, the remainder did not, which allows for the possibility that patients who had cleared their HCV infection or who had a biological false positive test, such as from an autoimmune disease, may have been incorrectly classified as having HCV. Despite this potential misclassification, a *post hoc* meta-analysis of studies that only included participants with HCV viremia in the HCV infection arm found no difference in HIV VL between HIV-HCV co-infected persons and persons with HIV mono-infection. The potential for misclassification in the other direction exists as well due to an increased risk for lack of anti-HCV antibody in chronically HCV-infected patients who are immunocompromised due to HIV infection [59]. However, the magnitude of this effect would likely be small; only approximately 5% of HIV-positive patients who are HCV antibody negative have been found to have detectable serum HCV RNA [59,60].

## Conclusions

In our systematic review, HCV infection was not associated with an increase in HIV VL among adults with HIV infection. This is in contrast to the substantial increases in HIV VL observed with several other systemic infections. Indeed, HCV infection appears to resemble HPV infection in that HIV accelerates progression of HCV disease without a reciprocal effect of HCV on HIV VL. Comparative analyses of the biological mechanisms underpinning HCV-HIV interactions and those responsible for interactions between HIV and other persistent or frequently recurrent infections such as herpes, malaria, TB and HPV offer critical opportunities to elucidate the complex pathways that lead to epidemiological synergy. Such analyses may ultimately enable us to predict which co-infections are most likely to accelerate the spread of HIV through populations and, conversely, which are most likely to have their own natural history or response to therapy adversely affected by HIV. Equally important, with the ongoing, rapid evolution of effective HCV therapies, HCV testing among HIV-infected individuals is recommended and should be aggressively implemented because of the increased rate of progression of liver disease in this population.

## Supplementary Material

Systematic review and meta-analysis of hepatitis C virus infection and HIV viral load: new insights into epidemiologic synergy
