# Supplementary material for: Systematic review and meta-analysis of hepatitis C virus infection and HIV viral load: new insights into epidemiologic synergy
Source: J Int AIDS Soc. 2016 Sep 19;19(1):20944. doi: 10.7448/IAS.19.1.20944 (PMC5030209; doi:10.7448/IAS.19.1.20944)

Supplementary Material

Systematic review and meta-analysis of HCV infection and HIV viral load: new insights into epidemiologic synergy

A post-hoc analysis of studies that only included patients with HCV viremia in the HIV-HCV co-infection arm [23-26, 29] also found that the mean log10 viral load did not differ between the HIV mono-infection and HIV-HCV co-infected patients (weighted mean difference (weighted mean difference -0.15, 95% CI -0.32 – 0.02, P=0.08; Fig.1).

Figure 1 Random effects meta-analysis of the mean difference in HIV viral load (log10 copies/mL) between HIV/HCV co-infected and HIV mono-infected individuals in studies documenting HCV viremia

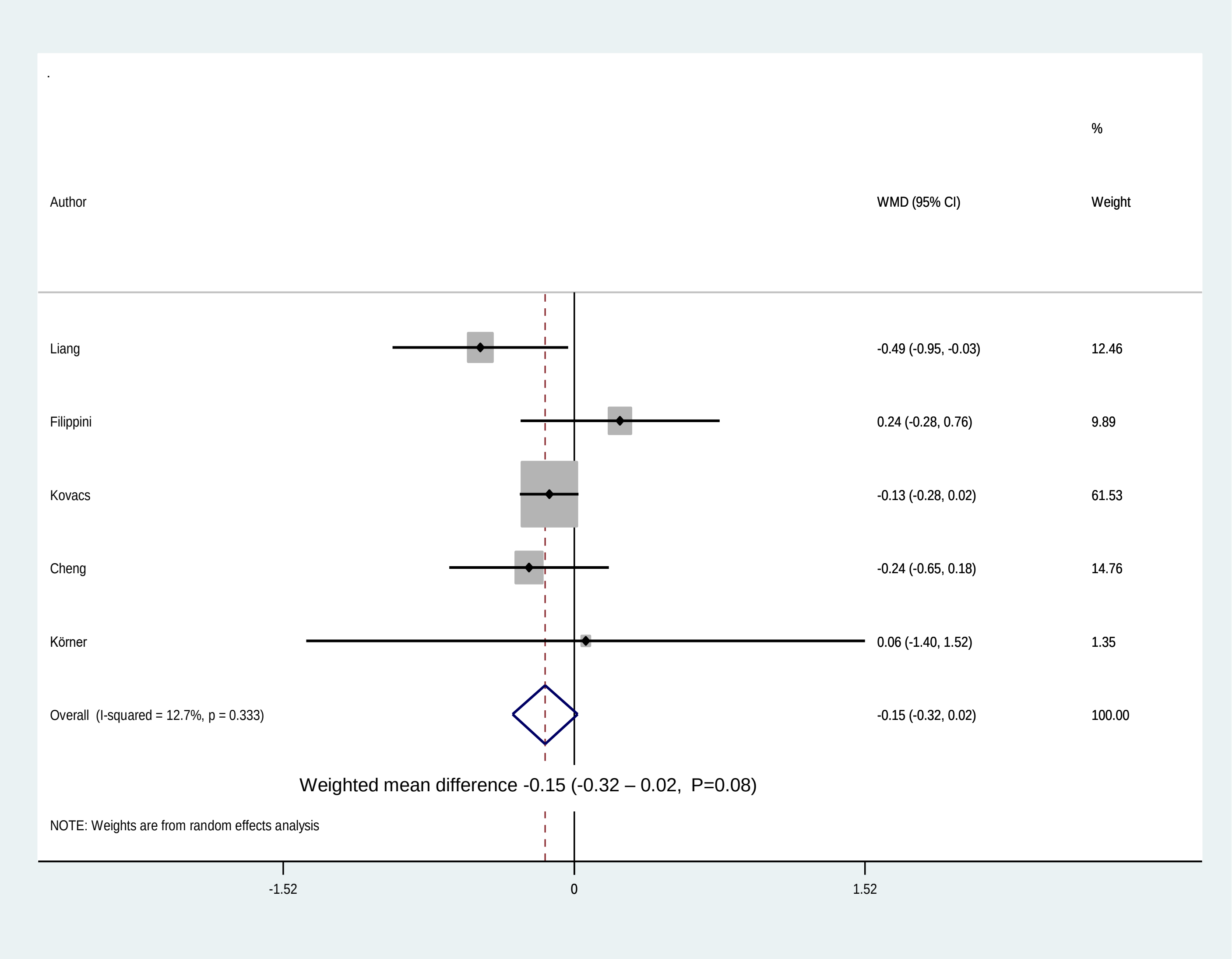

Supplement: Systematic review and meta-analysis of hepatitis C virus infection and HIV viral load: new insights into epidemiologic synergy [file JIAS-19-20944-s001.pdf]
